# Machine Learning Weather Soft-Sensor for Advanced Control of Wastewater Treatment Plants

**DOI:** 10.3390/s19143139

**Published:** 2019-07-17

**Authors:** Félix Hernández-del-Olmo, Elena Gaudioso, Natividad Duro, Raquel Dormido

**Affiliations:** 1 Department of Artificial Intelligence, National Distance Education University (UNED), Juan del Rosal 16, 28040 Madrid, Spain; 2 Department of Computer Sciences and Automatic Control, National Distance Education University (UNED), Juan del Rosal 16, 28040 Madrid, Spain

**Keywords:** wastewater treatment plants, soft-sensors, machine learning techniques

## Abstract

Control of wastewater treatment plants (WWTPs) is challenging not only because of their high nonlinearity but also because of important external perturbations. One the most relevant of these perturbations is weather. In fact, different weather conditions imply different inflow rates and substance (e.g., N-ammonia, which is among the most important) concentrations. Therefore, weather has traditionally been an important signal that operators take into account to tune WWTP control systems. This signal cannot be directly measured with traditional physical sensors. Nevertheless, machine learning-based soft-sensors can be used to predict non-observable measures by means of available data. In this paper, we present novel research about a new soft-sensor that predicts the current weather signal. This weather prediction differs from traditional weather forecasting since this soft-sensor predicts the weather conditions as an operator does when controling the WWTP. This prediction uses a model based on past WWTP influent states measured by only a few physical and widely applied sensors. The results are encouraging, as we obtained a good accuracy level for a relevant and very useful signal when applied to advanced WWTP control systems.

## 1. Introduction

Wastewater treatment has been one of the main objectives of the United Nations (UN) for years to guarantee the sustainability of the natural environment [1]. To guarantee an effective water treatment, much effort has been made to evaluate and reduce the impact of water treatment plants and to guarantee autonomous operation with the greatest possible energy savings.

One of the most demanding processes in a wastewater treatment plant (WWTP) is the active sludge process (ASP) with nitrification/denitrification stages [2]. Autonomous operation of WWTPs is based on the control of the values of certain variables for the good performance of the plant. In an ASP process, several variables are manipulated in WWTPs [3,4], for example, ammonia concentration or dissolved oxygen concentration (DO), which is one of the most widely used [5].

Several control strategies have been proposed to control DO concentration: PIDs (Proportional- Integral-Derivative) [6], Multivariable Control [7] or Predictive Multivariable Control [4,8].

Nevertheless, these methods do not adapt their operation to changes of the quality in load or flow. To adapt to these changes (mainly due to variations in the external weather conditions), plant operators manually operate the settings of these methods.

To provide more intelligent control, several approaches based on artificial intelligence techniques have been described in the literature, such as neural networks [7], support vector machines [9], regression [10], fuzzy logic [3] or genetic algorithms [11]. In a previous work [12], the authors proposed a reinforcement learning approach in a simulation model of the WWTP to reduce costs in the process. The reinforcement learning approach allows a quick and autonomous adaptation of the plant to changes in the environmental conditions with minimal intervention of the plant operator. More recently, the authors proposed [5] the use of a reinforcement learning agent with the goal of improving the energy and environmental efficiency for the N-ammonia removal process in WWTPs.

A common characteristic of all these control methods is that they require data about the characteristics of the water in the WWTP (temperature, soluble organic matter, oxygen, etc.) to operate efficiently. These data are usually obtained from physical sensors located at the plant.

However, many physical sensors are expensive to acquire and maintain. In addition, few of the physical sensors in WWTPs operate on-line [13]. Thus, several attributes of the water cannot be monitored on-line by means of physical sensors. In these cases, soft-sensors can provide on-line information that cannot be directly obtained from physical sensors. In fact, a soft-sensor is defined as a *model* that is capable of predicting variables that are hard to measure [14]. This model is built from previous data, called training data, obtained from physical sensors.

The output of a soft-sensor can be used for the on-line prediction of certain variables, process monitoring, process fault detection, or hardware-sensor monitoring [15]. Soft-sensors can be used to provide signals for a broad range of tasks depending on the available input data [15]. The prediction of certain output variables from data available in WWTPs is usually done by means of machine learning techniques. For example, artificial neural networks, feedforward neural networks or self-organizing maps have been used in the literature [15]. In addition, adaptive network-based fuzzy inference systems have been employed to develop models for the prediction of suspended solids [16]. A comprehensive review of different measures obtained by soft-sensors in WWTPs using machine learning techniques can be found in [15].

Plants operators are in charge of the process, and have to manage different settings of the plant depending on the different environmental conditions. One of the most relevant operational variables in WWTPs is the weather. However, weather is not an absolute measure. Weather is in some ways a subjective measure. There is an implicit uncertainty in how weather is perceived by different persons. The soft sensor designed in this paper for the prediction of current weather conditions (dry, rain or storm) is not an absolute weather sensor. It must learn from the best practices of plant operators what they consider a sufficient weather change to properly modify the set points. That is, the soft sensor learns the plant operator’s behavior. In other words, from the inflow data labeled by the operator, and using general machine learning techniques, the weather predictor is modeled with the final goal of improving the control of WWTPs.

To construct the soft-sensor, we completed the following steps that are common in a machine learning soft-sensor construction: data acquisition, data pre-processing, variable selection, model design, training and validation [15].

For the experiments, we used a widely known and common benchmark for the simulation of WWTPs: Benchmark Simulation Model 1 (BSM1) [17]. This benchmark is composed of an Active Sludge Model (ASM) [18]; the definition of the particular WWTP (number, dimensions and characteristics of the tanks, dimensions and characteristics of the clarifier, etc.); and, most important for this work, a dataset with most of the relevant characteristics of the influent (inflow wastewater) that arrives at the WWTP.

The rest of the paper is organized as follows. In the next section, we describe the machine learning techniques applied in the experimentation of the weather soft-sensor. Afterwards, we briefly describe BSM1 and its inflow dataset, which is followed by the exploration and pre-processing tasks performed on the dataset. In Section 3, we describe the results obtained in the experiments. We conclude in Section 4 with a discussion of the results.

## 2. Materials and Methods

In this section, we begin with a description of the machine learning methods we used to generate the weather soft-sensor signal. Afterwards, we briefly explain the WWTP plant, called BSM1, from which we obtained the inflow dataset. Next, we show the details of the variables of the influent. Finally, we explore the dataset and explain the pre-processing we applied to obtain the results presented in Section 3.

### 2.1. Machine Learning for Soft-Sensors in WWTPs

Many applications use soft-sensors in industrial process control because they can improve the quality of the product and guarantee the safety of the process.

In this study, we used different machine learning techniques to model a soft-sensor to predict weather conditions such as Support Vector Machine, k-nearest neighbors, Decision Trees, Random Forest and Gaussian Naive Bayes. All methods were implemented in the R [19] framework. In the next subsections, we show how these techniques work and, in particular, how they operate in WWTPs.

There are many examples of the use of these machine learning techniques for modeling soft-sensors (e.g., [20,21]). Specifically, these techniques have been used successfully in WWTPs, as  shown below.

#### 2.1.1. Support Vector Machines

Support Vector Machine (SVM) is a binary supervised classification algorithm [22]. The SVM model represents the data in space, separating the classes into two spaces that are as wide as possible through a hyperplane called the support vector. The success rate of SVM is especially high when the training dataset is good enough. The results obtained in this study are proof of this. SVM is widely applied to soft-sensor models and also in WWTPs [23,24].

#### 2.1.2. K-Nearest Neighbors

K-Nearest Neighbors (KNN) is also a supervised algorithm used for classification and regression [25]. It is a simple method used to classify a dataset by only looking at the most similar data points (by proximity) learned in the training stage. Then, when a new dataset is classified, it is assigned to the most common dataset among its *k* nearest neighbors (where *k* is a small positive integer). This technique has many applications using soft-sensors [26] as well as in WWTPs [27].

#### 2.1.3. Decision Trees

A decision tree is a supervised classification algorithm [28] that recursively partitions a dataset into smaller sets, based on a set of tests defined in each node of the tree. The tree has a root node formed from all the initial data, a set of intermediate nodes resulting from the divisions and a set of terminal nodes, called leaves. Decision trees do not require assumptions regarding the distributions of the input data. There are many examples of the use of decision tree with soft-sensors [29] as well as in WWTPs [30].

#### 2.1.4. Random Forest

Random Forest is a supervised classification algorithm [31] that generates a set of classification or regression trees in a different way from a conventional decision tree algorithm (see above). Therefore, in addition to building each tree with a different sample of the data, the RF algorithm changes the way trees are constructed. With RF, each node of the tree is divided using the best possible tree among a subset of predictors or features selected at random in that node. Therefore, the search processes of the root node and the division of the feature nodes are executed randomly. There are many examples of the use of RF with the soft-sensor [32,33] as well as in WWTPs [34].

#### 2.1.5. Gaussian Naive Bayes

A Gaussian Naive Bayes classifier [35] is a probabilistic classifier based on Bayes’ theorem that considers there is independence between the predictor variables. In other words, it assumes that the presence or absence of a feature is not related to the presence or absence of any other characteristic. Each characteristic contributes independently to the probability that a datum belongs to a set, independently of the presence or absence of the other characteristics. These classifiers can be trained efficiently in a supervised learning environment, since they do need many data to estimate the necessary parameters for the classification. They are widely used in the literature, specifically in systems that use soft-sensors [36] as well as in WWTPs [37].

### 2.2. WWTP Benchmark Simulation Model 1

For the experiments, we used data from the known WWTP benchmark BSM1 [17]. BSM1 is a simulation environment that defines a plant layout incorporating an active sludge model, influent loads, test procedures and evaluation criteria.

**Figure 1:**
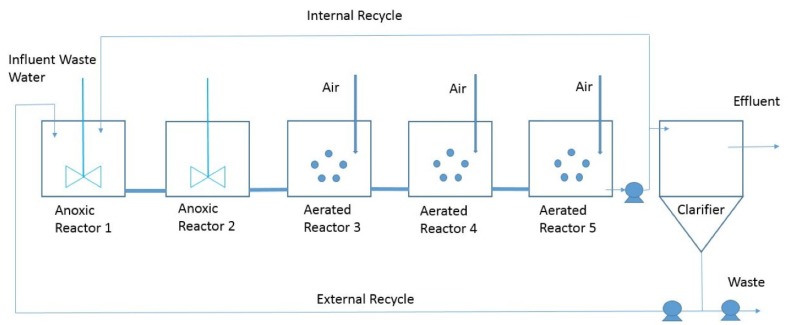
Plant of the Benchmark Simulation Model 1 (BSM1).

In BSM1, the plant is a five-compartment activated sludge reactor. The plant has two anoxic tanks followed by three aerobic tanks (see Figure 1). Therefore, the plant combines nitrification with denitrification using a configuration that it is often used to achieve biological nitrogen removal in full-scale plants [38].

More details about Figure 1 can be found in [5].

### 2.3. Exploration and Pre-Processing of BSM1 Inflow Data

The dataset used in our experiments is part of BSM1 [39]. In BSM1, the inflow wastewater characteristics through time are collected into three input data files, one file for each weather conditions we considered in this study: dry, rain and storm events. These input data were collected for two weeks of operation and in 15-min intervals. The attributes that characterize the influent are shown in Table 1. Each row in each dataset corresponds to a measure of these attributes every 15 min. In this study, we only used the second week of each file.

In a real environment, these attributes cannot be measured directly from sensors in water [40,41]. Moreover, it is difficult and expensive to measure all of these attributes every 15 min. Thus, we focused on only a few measures that are more easily obtained from real physical sensors: Q (inflow rate), COD (chemical oxygen demand), $BOD_{5}$ (five-day biochemical oxygen demand), N-ammonia (ammonia concentration) and N-Kjedahl (amount of nitrogen for denitrification) [2].

To work with these sensors in our experiment, we transformed the BSM1 inflow dataset using Equations (1)–(4). The constants $f_{p}$ (endogenous residue), $i_{xb}$ (nitrogen content of active mass) and $i_{xp}$ (nitrogen content of endogenous mass) characterize the BSM1 plant [17].

(1) $$BOD_{5}=0.65*\left(S_{s}+X_{s}+\left(1-f_{p}\right)*\left(X_{bh}+X_{ba}\right)\right)$$

(2) $$COD=S_{s}+S_{i}+X_{s}+X_{i}+X_{bh}+X_{ba}+X_{p}$$

(3) $$N\_ammonia=S_{nh}$$

(4) $$N\_Kjedahl=S_{nh}+S_{nd}+X_{nd}+i_{xb}*\left(X_{bh}+X_{ba}\right)+i_{xp}*\left(X_{p}+X_{i}\right)$$

First, we explored the correlation among these measures to detect redundancies as fewer sensors leads to cheaper and less complex systems. In Table 2, we can see that COD and $BOD_{5}$ are extremely correlated. In addition, N-ammonia and N-Kjedahl are very correlated. Therefore, among the physical sensors considered, finally we only selected Q, COD and N-ammonia. In fact, these sensors are affordable on-line sensors, and becoming increasingly common in WWTPs [41]. In addition, this selection also freed the machine learning algorithms from redundant attributes that would have made their job harder.

**Figure 2:**
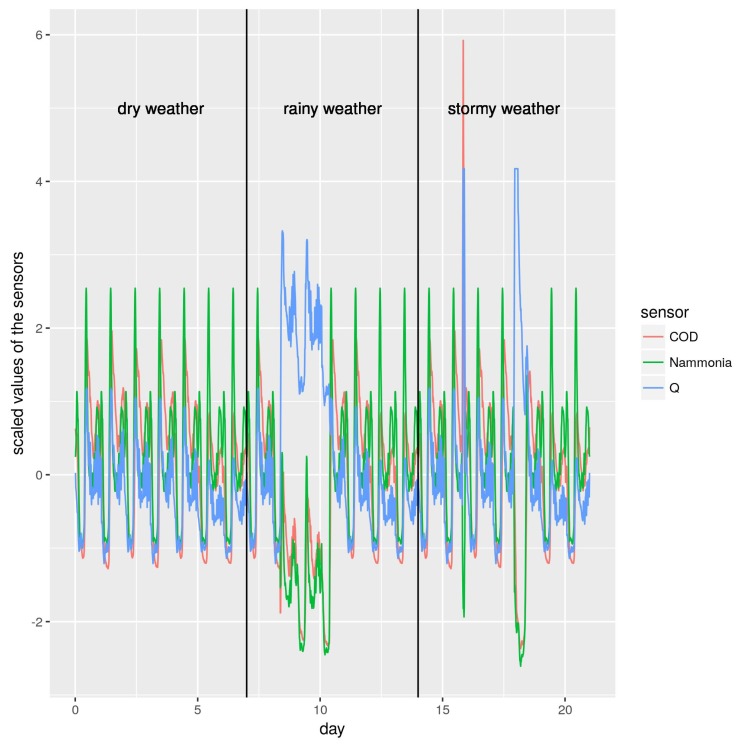
Relation among Q, COD and N-ammonia measures after been scaled.

Next, we explored the transformed data only measured by Q, COD and N-ammonia sensors. In Figure 2, we can see the behavior of these three values through the three labeled weather conditions: dry weather, rainy weather and stormy weather. All variables were scaled in the same way using a standard technique to obtain more uniform data. In this scale, for each variable *x*, the distribution mean and standard deviation were calculated, which were then normalized with zero-mean and unit-variance using Equation (5).

(5) $$x_{norm}=\frac{x-\hat{x}}{\sigma_{x}}$$

where $\hat{x}$ is the mean and $\sigma_{x}$ is the standard deviation. It can be seen in Figure 2 that there are many instants of time with similar values, despite being different weather conditions (for instance, on Days 6, 13 and 20). This fact made this task harder for the machine learning algorithms, as shown in Section 3.

To break the similarity among values of different weather conditions, we considered values of the sensor that are close in time. To this end, we decided to apply a first-order lag filter [42] to every sensor and use these filter outputs as new attributes for the machine learning algorithms. The filtered signal f(t) was calculated as shown in Equation (6).

**Figure 3:**
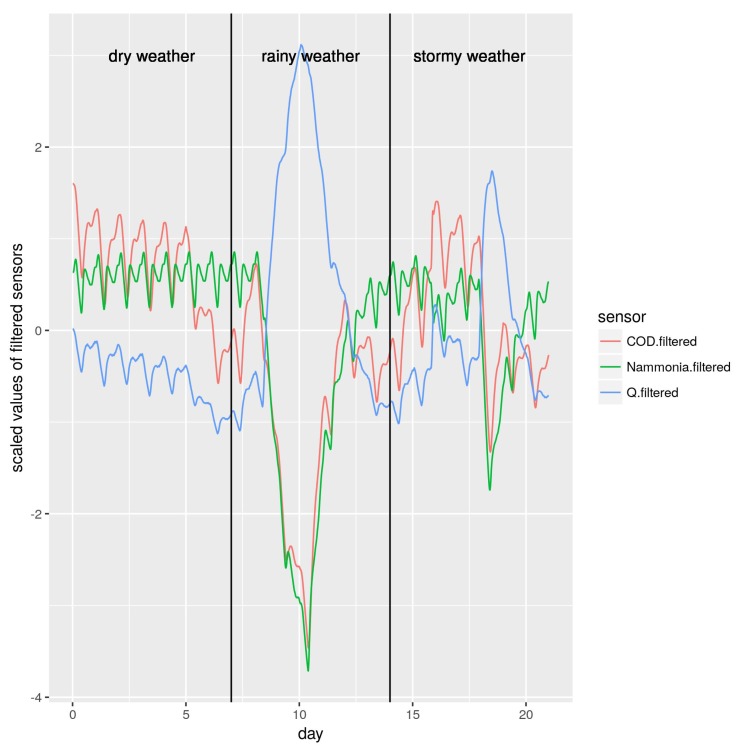
Relationships among Q, COD and N-ammonia measures after being scaled and filtered.

(6) f(t)=α∗f(t−1)+(1−α)∗s(t)

where s(t) is the measured of the sensor and α is the filter constant. The bigger α is, the stronger is the filter, being α=0 when no filter is applied. The time constant is 15 min, the sampling time in the dataset. In Figure 3, we show the values of these three filtered measures. Now, the values of the three sensors could be used more easily to characterize and differentiate each weather condition. In addition, notice that values were scaled. This helped both the visualization and the machine learning algorithms.

**Figure 4:**
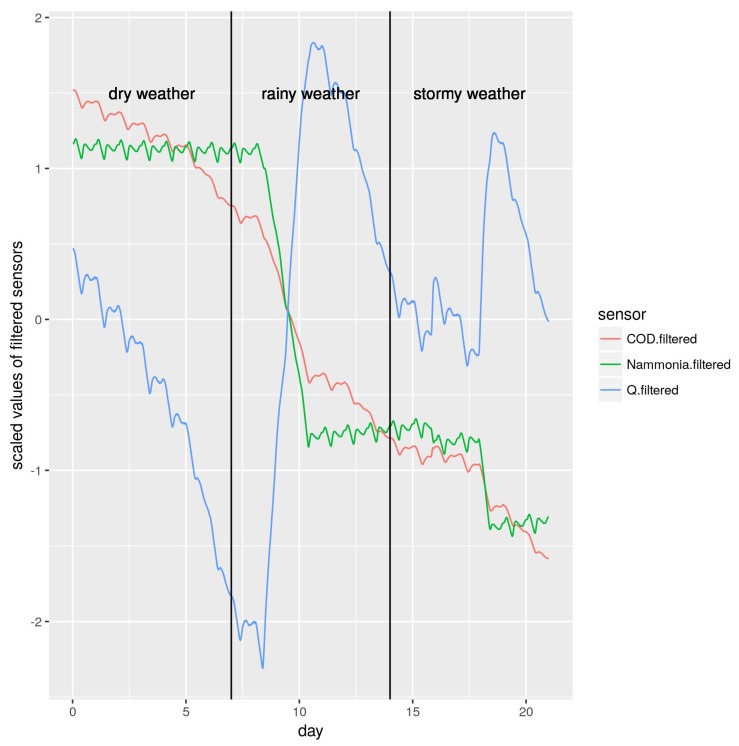
Relationship among Q, COD and N-ammonia measures after bring scaled and strongly filtered.

Finally, to explore how each filtered value changed the sensors’ performance, we also added a strong filter so that we could compare the effects of too much filtering. The effects of applying a strong filter on the three signals are shown in Figure 4. Now, the values of the three sensors could be easily used to differentiate each weather condition. At  first sight, it appears this should make the prediction task easier. However, we shown in Section 3 that this is not the case.

## 3. Results

In this section, we use the previously described data to feed the machine learning algorithms, so that our soft-sensor can learn to predict the weather condition signal. To this end, the machine learning algorithms described in Section 2 were used.

The training dataset was built using three weeks of data in a row: seven days of dry weather, seven days of rainy weather, and seven days of stormy weather. To evaluate results, we measured accuracy in the following two ways:

(i) traditional 10-fold-cross validation over the inflow dataset; and
(ii) a validation dataset after the training dataset, where the machine learning algorithms first learned the model through a training dataset and then the models were applied on a validation dataset to predict the weather signal.

### 3.1. 10-Fold-Cross Validation

As explained in Section 2, we ran three kinds of experiments: (i) no filter; (ii) smooth filter; and (iii) strong filter. Results are shown in Table 3. In the strong filter row, we obtained outstanding accuracy rates. This was mainly caused by an overfit to the training data, as probed in the following validation phase. Moreover, in Figure 4, we can see that we obtained the most distinct values for each weather condition, helping the machine learning algorithms in their task. If we had only this environmental condition, results would be great with this kind of filter. However, WWTPs can experience dry, rainy or even stormy events without any previous notice after the training phase. Thus, we show results in the next subsection with different validation datasets after the training phase.

To this end, we decided to evaluate with a validation dataset after the training phase. Thus,  we first created the *training dataset* by concatenating the three datasets dry–rain–storm again as in Figure 2. Secondly, we created $3^{3}$ *validation datasets* by concatenating all combinations of the three weather conditions: dry–dry–dry, dry–dry–rain, dry–dry–storm, …, storm–storm–rain, and storm–storm–storm. To illustrate the process, we show in Table 4 the particular combination rain–dry–storm as an instance example. Finally, in Table 5, we show the mean accuracy of the 27 validation datasets. Notice that, for each evaluation, we had to concatenate training data and validation data so that filters could be applied.

### 3.2. Validation Dataset

As shown in the last subsection, we need a more realistic evaluation approach to assess well our weather soft-sensor.

Finally, in Table 6, we show the correlation between measures from physical sensors and the soft-sensor data from the best classifiers. Notice that now they were calculated from the validation datasets, not from the training dataset as in Table 2, thus there are small differences. Here, when we focus on correlations between the weather soft-sensor and the physical sensors, we see almost no correlations at all. In fact, the most correlated measures are between the two weather soft-sensors, which makes sense.

## 4. Discussion

In this work, we sought a soft-sensor that informs the advanced control system of a WWTP about the current weather condition by means of the inflow characteristics. The current weather signal is really important to improve the advanced control system in a WWTP. To this end, we wanted the inflow variables to be measured by as few widely applied sensors as possible. As discussed in Section 2, we ended up with just three widely used sensors: Q, COD and N-ammonia.

We applied machine learning techniques to predict the current weather conditions from these three sensors. However, the current weather conditions experienced by the WWTP is not an absolute measure and it depends on the perception and the previous experiences of the operator in the plant. In fact, the plant operator perception of weather conditions is focused on the control of the plant so the characteristics for a dry, rainy or stormy weather may differ from a traditional weather forecast. Thus, the weather soft-sensor must *learn* what the WWTP plant operator considers dry, rainy or stormy weather for an efficient control of the plant. In our opinion, this is the main reason we can see similar measures of Q, COD and N-ammonia under different weather conditions (see Figure 2). The last implies that a raw consideration of sensors output makes this problem a really difficult task for machine learning predictors (see Section 3 and Table 3 and Table 5).

To break this similarity of measures, in the pre-processing phase, we applied a first-order lag filter. However, if the filter were too strong, this breaking would be too high, which would overfit the machine learning model. Therefore, as shown in Section 3 (Table 3), we obtained high accuracy measures when applying a strong filter that had to be discarded when assessing an experiment with a more realistic validation dataset (see Table 5).

Finally, we obtained an approximately 85% accuracy in the weather soft-sensor with two machine learning algorithms: KNN(1) and Random Forests. These results are encouraging, thus, as future work, it is intended to demonstrate the performance of the more accurate soft sensors to tackle advanced control tasks in WWTPs process. For instance, our previous results [5,43] could be improved by using these sensors. The real plant where we will test these sensors are the raceways reactors located at the IFAPA Research Center (Almería, Spain). This pilot plant belongs to the project that financed this work.

## Figures and Tables

**Table 1:** Attributes that characterize the inflow in the BSM1 plant.

Attribute	Definition
Flowrate	Q
Soluble inert organic matter	$S_{I}$
Readily biodegradable substrate	$S_{S}$
Particulate inert organic matter	$X_{I}$
Slowly biodegradable substrate	$X_{S}$
Active heterotrophic biomass	$X_{B,H}$
Active autotrophic biomass	$X_{B,A}$
Particulate products arising from biomass decay	$X_{P}$
Oxygen	$S_{O}$
Nitrate and nitrite nitrogen	$S_{NO}$
$NH4^{+}+NH_{3}$ Nitrogen	$S_{NH}$
Soluble biodegradable organic nitrogen	$S_{ND}$
Particulate biodegradable organic nitrogen	$X_{ND}$
Alkalinity	$S_{ALK}$

**Table 2:** Correlation among real sensors on all weather data.

	Q	BOD5	COD	N_Kjedahl	N_Ammonia
Q	1.00	−0.07	0.03	−0.07	−0.19
BOD5	−0.07	1.00	0.99	0.91	0.74
COD	0.03	0.99	1.00	0.89	0.70
N_Kjedahl	−0.07	0.91	0.89	1.00	0.94
N_ammonia	−0.19	0.74	0.70	0.94	1.00

**Table 3:** 10-fold-crossvalidation accuracy on training dataset (dry+rain+storm).

Experiment	Naive.Bayes	Decision.Tree	KNN(1)	KNN(3)	Random.Forest	SVM
no filter	0.41	0.44	0.14	0.17	0.14	0.44
smooth filter	0.56	0.80	0.79	0.83	0.95	0.73
strong filter	0.88	1.00	0.99	0.99	1.00	0.98

**Table 4:** Validation for a paticular combination of weather condition rain-dry-storm.

Experiment	Naive.Bayes	Decision.Tree	KNN(1)	KNN(3)	Random.Forest	SVM
no filter	0.41	0.45	0.46	0.47	0.47	0.45
smooth filter	0.55	0.72	0.78	0.76	0.78	0.65
strong filter	0.24	0.33	0.36	0.36	0.33	0.33

**Table 5:** Accuracy mean of the $3^{3}$ validation datasets.

Experiment	Naive.Bayes	Decision.Tree	KNN(1)	KNN(3)	Random.Forest	SVM
no filter	0.41	0.45	0.46	0.46	0.47	0.45
smooth filter	0.56	0.75	0.85	0.82	0.84	0.68
strong filter	0.39	0.33	0.35	0.35	0.33	0.33

**Table 6:** Final correlation between measures and predictions of the best two classifiers.

	Q	COD	N_Ammonia	KNN(1)	Random.Forest
Q	1.00	0.05	−0.16	0.05	0.08
COD	0.05	1.00	0.69	−0.00	−0.00
N_ammonia	−0.16	0.69	1.00	−0.03	−0.06
KNN(1)	0.05	−0.00	−0.03	1.00	0.67
Random.Forest	0.08	−0.00	−0.06	0.67	1.00

## References

[1] Nations U. (2015). The Millennium Development Goals Report.

[2] Metcalf E., Eddy H. (2003). Wastewater Engineering: Treatment and Reuse.

[3] Yang T., Qiu W., Ma Y., Chadli M., Zhang L. (2014). Fuzzy model-based predictive control of dissolved oxygen in activated sludge processes. Neurocomputing.

[4] Holenda B., Domokos E., Redey A., Fazakas J. (2008). Dissolved oxygen control of the activated sludge wastewater treatment process using model predictive control. Comput. Chem. Eng..

[5] Hernandez-del Olmo F., Gaudioso E., Dormido R., Duro N. (2016). Energy and Environmental Efficiency for the N-Ammonia Removal Process in Wastewater Treatment Plants by Means of Reinforcement Learning. Energies.

[6] Hamitlon R., Braun B., Dare R., Koopman B., Svoronos S. (2006). Control issues and challenges in wastewater treatment plants. IEEE Control Syst. Mag..

[7] Han H., Qiao J., Chen Q. (2012). Model predictive control of dissolved oxygen concentration based on a self-organizing RBF neural network. Control Eng. Pract..

[8] Revollar S., Vega P., Vilanova R. Economic optimization of Wastewater Treatment Plants using Non Linear Model Predictive Control. Proceedings of the 2015 19th International Conference on System Theory, Control and Computing (ICSTCC).

[9] Ribeiro D., Sanfins A., Belo O., Perner P. (2013). Wastewater Treatment Plant Performance Prediction with Support Vector Machines. Advances in Data Mining. Applications and Theoretical Aspects.

[10] Yu Y., Zou Z., Wang S. (2019). Statistical regression modeling for energy consumption in wastewater treatment. J. Environ. Sci..

[11] Bagheri M., Mirbagheri S., Bagheri Z., Kamarkhani A. (2015). Modeling and optimization of activated sludge bulking for a real wastewater treatment plant using hybrid artificial neural networks-genetic algorithm approach. Process Saf. Environ. Prot..

[12] Hernandez-del Olmo F., Gaudioso E. (2012). An Emergent Approach for the control of WasteWater Treatment Plants by means of reinforcement learning techniques. Expert Syst. Appl..

[13] Vanrolleghem P., Lee D. (2003). On-Line Monitoring Equipment for Wastewater Treatment Processes: State of the Art. Water Sci. Technol..

[14] Alexandridis A. (2013). Evolving RBF neural networks for adaptive soft-sensor design. Int. J. Neural Syst..

[15] Haimi H., Mulas M., Corona F., Vahala R. (2013). Data-derived soft-sensors for biological wastewater treatment plants: An overview. Environ. Model. Softw..

[16] Thürlimann C., Dürrenmatt D., Villez K. (2018). Soft-sensing with qualitative trend analysis for wastewater treatment plant control. Control Eng. Pract..

[17] Copp J. (2002). The COST Simulation Benchmark: Description and Simulator Manual.

[18] Henze M., Gujer W., Mino T., Loosdrecht M.V. (2000). Activated Sludge Models ASM1, ASM2, ASM2d and ASM3.

[19] R Core Team (2013). R: A Language and Environment for Statistical Computing.

[20] Kadlec P., Gabrys B., Strandt S. (2009). Data-driven Soft Sensors in the process industry. Comput. Chem. Eng..

[21] He Y., Geng Z., Zhu Q. (2015). Data driven soft sensor development for complex chemical processes using extreme learning machine. Chem. Eng. Res. Des..

[22] Vapnik V.N. (1998). Statistical Learning Theory.

[23] Liu G., Zhou D., Xu H., Mei C. (2010). Model optimization of SVM for a fermentation soft sensor. Exp. Syst. Appl..

[24] Guo H., Jeong K., Lim J., Jo J., Kim Y., Park Y., Kim J., Cho K. (2015). Prediction of effluent concentration in a wastewater treatment plant using machine learning models. J. Environ. Sci..

[25] Altman N.S. (1992). An Introduction to Kernel and Nearest-Neighbor Nonparametric Regression. Am. Stat..

[26] Kaneko H., Funatsu K. (2013). Estimation of predictive accuracy of soft sensor models based on data density. Chemom. Intell. Lab. Syst..

[27] Kim M., Kim Y., Kim H., Piao W., Kim C. (2016). Evaluation of the k-nearest neighbor method for forecasting the influent characteristics of wastewater treatment plant. Front. Environ. Sci. Eng..

[28] Breiman L., Friedman J.H., Olshen R.A., Stone C.J. (1984). Classification and Regression Trees.

[29] Xu M., Watanachaturaporn P., Varshney P.K., Arora M. (2005). Decision tree regression for soft classification of remote sensing data. Remote Sens. Environ..

[30] Carrasco E., Rodríguez J., Puñal A., Roca E., Lema J. (2002). Rule-based diagnosis and supervision of a pilot-scale wastewater treatment plant using fuzzy logic techniques. Exp. Syst. Appl..

[31] Breiman L. (2001). Random Forests. Mach. Learn..

[32] Pal M. (2005). Random forest classifier for remote sensing classification. Int. J. Remote Sens..

[33] Pardo M., Sberveglieri G. (2008). Random forests and nearest shrunken centroids for the classification of sensor array data. Sens. Actuators B Chem..

[34] Torregrossa D., Schutz G., Cornelissen A., Hernández-Sancho F., Hansen J. (2016). Energy saving in WWTP: Daily benchmarking under uncertainty and data availability limitations. Environ. Res..

[35] Cooper G.F., Herskovits E. (1992). A Bayesian method for the induction of probabilistic networks from data. Mach. Learn..

[36] Yan W., Shao H., Wang X. (2004). Soft sensing modeling based on support vector machine and Bayesian model selection. Comput. Chem. Eng..

[37] Li D., Yang H., Liang X.F. (2013). Prediction analysis of a wastewater treatment system using a Bayesian network. Environ. Model. Softw..

[38] Gernaey K., Jeppsson U., Vanrolleghem P., Copp J. (2014). Benchmarking of Control Strategies for Wastewater Treatment Plants.

[39] Alex J., Benedetti L., Copp J., Gernaey K., Jeppsson U., Nopens I., Pons M., Rieger L., Rosen C., Steyer J. Benchmark Simulation Model no. 1 (BSM1). https://www.iea.lth.se/publications/Reports/LTH-IEA-7229.pdf.

[40] Smith R. Chemical Oxygen Demand in Influent Wastewater Monitoring. https://www.ysi.com/ysi-blog/water-blogged-blog/2017/01/chemical-oxygen-demand-in-influent-wastewater-monitoring.

[41] Rhosonics The Benefits of in-Line COD Measurement in Industrial Wastewater. https://www.rhosonics.nl/news/the-benefits-of-in-line-cod-measurement-in-industrial-wastewater/.

[42] Ogata K. (2010). Modern Control Engineering.

[43] Hernández-del Olmo F., Gaudioso E., Dormido R., Duro N. (2018). Tackling the start-up of a reinforcement learning agent for the control of wastewater treatment plants. Knowl.-Based Syst..

